# Electronic Information Standards to Support Obesity Prevention and Bridge Services Across Systems, 2010–2015

**DOI:** 10.5888/pcd14.160299

**Published:** 2017-10-26

**Authors:** Jennifer L. Wiltz, Heidi M. Blanck, Brian Lee, S. Lawrence Kocot, Laura Seeff, Lisa C. McGuire, Janet Collins

**Affiliations:** 1 Economic Studies, Center for Health Policy, Brookings Institution, Washington, DC; 2 Division of Nutrition, Physical Activity, and Obesity, National Center for Chronic Disease Prevention and Health Promotion, Centers for Disease Control and Prevention, Atlanta, Georgia; 3 Office of Public Health Scientific Services, Centers for Disease Control and Prevention, Atlanta Georgia; 4 Office of Health Systems Collaboration, Office of the Associate Director of Policy, Office of the Director, Centers for Disease Control and Prevention, Atlanta, Georgia

## Abstract

Electronic information technology standards facilitate high-quality, uniform collection of data for improved delivery and measurement of health care services. Electronic information standards also aid information exchange between secure systems that link health care and public health for better coordination of patient care and better-informed population health improvement activities. We developed international data standards for healthy weight that provide common definitions for electronic information technology. The standards capture healthy weight data on the “ABCDs” of a visit to a health care provider that addresses initial obesity prevention and care: **a**ssessment, **b**ehaviors, **c**ontinuity, i**d**entify resources, and **s**et goals. The process of creating healthy weight standards consisted of identifying needs and priorities, developing and harmonizing standards, testing the exchange of data messages, and demonstrating use-cases. Healthy weight products include 2 message standards, 5 use-cases, 31 LOINC (Logical Observation Identifiers Names and Codes) question codes, 7 healthy weight value sets, 15 public–private engagements with health information technology implementers, and 2 technical guides. A logic model and action steps outline activities toward better data capture, interoperable systems, and information use. Sharing experiences and leveraging this work in the context of broader priorities can inform the development of electronic information standards for similar core conditions and guide strategic activities in electronic systems.

## Introduction

The overall age-adjusted prevalence of obesity among US adults aged 20 years or older in 2013–2014 was approximately 38% (1); obesity’s effects on health, including its contributions to chronic disease and disability, underscore the need to strengthen prevention efforts in the United States (2). Best practices for preventing and managing obesity include providing care per the Chronic Care Model, empowering patients for self-management and bridging primary care activities and public health efforts (3,4). Innovations in electronic health information (EHI) (Box) can support these best practices. EHI consists of electronic health records (EHRs), personal health records, personal wellness devices, health information exchanges, registries, and population health databases, all of which can support health systems, patients, communities, and public health practitioners in achieving individual health and population health. The collection of weight-related data and the development of specialized registries are priorities in policies (5) and initiatives (6–9) that can be applied to promote healthy behaviors and prevent diseases such as obesity, heart disease, stroke, cancer, and diabetes.

**Box. Glossary of Information Technology (IT) Terms**

*Clinical document architecture (CDA)*: a standard developed by Health Level 7 International (HL7) that defines the data structure and semantics (ie, interpretation) of clinical documents, such as discharge summaries and progress notes, to facilitate better electronic data exchange.

*Data element*: a unit of data defined (such as by character length or type) for processing; multiple elements are combined into a patient record or document.

*Electronic health information (EHI)*: information in a digital format that is captured or transferred by computerized tools such as electronic health records (EHRs), personal health records, personal wellness devices, health information exchanges, registries, and population health databases.

*Electronic health record (EHR)*: a computerized collection of patient and population health information that is stored in a digital format and maintained by health professionals and official agencies.

*Electronic information standards*: specifications established by a standardizing organization that define and enhance electronic information management by describing structure, encoding, and meaning. Examples are message content and interoperability requirements.

*Harmonization*: adjustment of differences and inconsistencies among items, such as specifications, methods, or systems, to make them aligned, uniform, or mutually compatible across organizations or entities.

*Healthy Weight message*: a communication, such as a collection of content or codes such as those defined for the Healthy Weight standard, that is sent electronically from one IT tool to another in an exchange of electronic information; the sender and receiver of this information must both be similar, or built to the same standard, in order for the interaction to be successful.

*Profile*: a document developed by Integrating Healthcare Enterprise International (IHE) that provides a common language for purchasers, vendors, and developers and defines precisely how standards (for example, in this article, Healthy Weight standards) can be implemented to meet certain clinical needs.

*Healthy Weight standards*: specifications related to healthy weight that enhance electronic information management.

*Healthy Weight visit*: a health care provider visit that addresses the first steps of obesity prevention and management in a stepwise approach to care as well as chronic disease prevention; this initial health promotion and planning could be applicable to the overall patient population (whereas subsequent follow up, if indicated, would provide more in-depth management).

*Information technology (IT) implementer*: a person who puts a system into practice. In this article on Healthy Weight standards, implementers fulfill or build the electronic system to specifications so they can send or receive healthy weight messages.

*Interoperability*: the ability to securely and seamlessly exchange, interpret, and use information among permitted information systems.

*Interoperability standard*: specifications that enable data to be shared among those with permissions (eg, clinician, hospital, public health, patient, laboratory, pharmacy) regardless of the application or application vendor.

*Logical observation identifiers names and codes (LOINC)*: a database of standardized codes for medical observations.

*Message standard*: specifications that define the content (eg, codes) and structure of a message.

*Object identifier*: a globally unambiguous, unique, persistent name assigned to something in computing, such as the unique number given to the value sets housed in the PHIN VADS (Public Health Information Network Vocabulary Access and Distribution System).

*Observational result (ORU)*: a Health Level Seven International (HL7) message standard that provides structured patient-oriented data on clinical observations.

*Use-case scenario*: a sequence of event steps depicting how data can meaningfully flow and be used by people (eg, clinician, patient, pharmacist, public health worker) across systems.

*Standard: *a specification that is an established norm or requirement that describes how to define, structure, and organize data or information.

*Systematized Nomenclature of Medicine (SNOMED)*: a standardized vocabulary of clinical terminology that is used by physicians and other health care providers for the electronic exchange of clinical health information.

*Value set*: a group of codes used to define clinical concepts.

The collection of EHI can support health care providers in delivering obesity-related care through screening, counseling, goal setting, and care planning. This information in turn can help health care providers identify, document, and manage obesity, which can improve health behaviors (10–13). Many physicians report that their EHR cannot calculate body mass index (BMI) or pediatric BMI percentile (14), both of which are routinely needed at the outset of a clinical visit (15,16). Moreover, less than 10% of health care providers reported having advanced EHR functions, such as the ability to link patients to resources after a clinic visit (14). Additionally, EHI systems that allow for selected information to be securely and seamlessly exchanged among permitted systems, a capability termed *interoperability*, are useful for coordinating patient care, reporting data to public health agencies, and conducting population health analytics (7,17,18). For example, referral information can be exchanged among a primary physician, a patient navigator, and a dietitian. EHI-based surveillance data can be used by public health agencies to inform and prioritize community-based prevention activities. Data analyzed by public health agencies could then be cycled back to health care providers to help them counsel patients and improve their clinical practice. Multiple approaches for sharing data, such as dual data entry or multiple interfaces, can be time intensive and costly, preclude efficient communication processes, and increase the risk of inconsistent, incomplete data (19).

Methods for conducting population health analytics are limited to single systems or sectors or are lacking in one or more characteristics that allow for ideal use, or both (20). For example, some surveillance data are collected according to strict measurement protocols (eg, measured weight in the National Health and Nutrition Examination Survey [NHANES]), but the data do not provide state-level estimates. In contrast, other systems provide state-level data, but the data are not as valid as the data in NHANES (eg, self-reported weight in the Youth Risk Behavior Survey). National and state data may not provide the details needed by local entities to target interventions to the characteristics of their communities. Ideally, information systems would provide uniform, timely, high-quality, objectively measured data at the local level (eg, counties, health care providers’ offices), and these data would accurately represent various population subgroups (eg, the population of children, which has larger gaps in BMI surveillance data than does the population of adults). To enhance population health analytics, some organizations have initiated independent EHI-based surveillance systems that collect obesity-related data at the local level (19). However, these organizations have reported challenges in obtaining and exchanging uniform, high-quality data. These challenges resulted from a lack of standards in international message content and interoperability and create difficulties in using information optimally to fill gaps in prevention efforts (19).

We describe the development of electronic information standards for weight measures across EHI systems. Our objective was to develop healthy weight standards that can be used in the United States and globally to support the collection of uniform, high-quality data to improve the delivery of health care and the exchange of information to benefit patients, clinicians, health systems, and communities. These newly developed standards were named Healthy Weight standards.

## Methods for Creating Standards for Multiple Sectors

**Figure 1:**
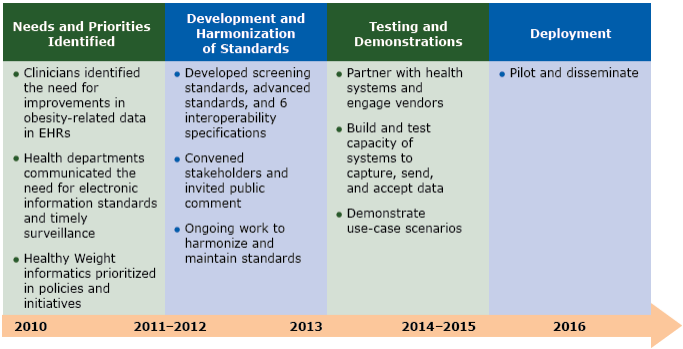
Process for creating Healthy Weight standards**. **All activities were undertaken in collaboration with stakeholders: state and local partners (via webinars), the Healthy Weight EHR Expert Panel, professional academies, and information technology (IT) vendors. Abbreviations: EHR, electronic health record.

Key steps in the process of developing the Healthy Weight standards were the following: identifying needs and priorities for electronic information standards; developing and aligning standards for data content and interoperability; testing and demonstrating data transmission; and deploying the standards (Figure 1). These activities followed other processes that enable creation of harmonized (ie, aligned so that they are similar across organizations or entities) standards, interoperability specifications, and implementation guidance (www.siframework.org/framework.html; http://phdsc.org/standards/health-information-tech-standards.asp).

Clinicians identified the need for improvements in obesity-related data in the EHR, and public health practitioners communicated the need for electronic information standards (19). Additionally, informatics (ie, information processing and systems engineering) has been prioritized in policies (5) and initiatives (6–9). These needs and priorities prompted the Centers for Disease Control and Prevention (CDC) to begin creating message standards and interoperability standards. Stakeholders (eg, health care providers, health information technology [IT] vendors, public health professionals, federal agencies, international organizations) engaged in the process of defining the priorities and the scope of the Healthy Weight data elements informed the initial steps, or “ABCDs,” of obesity-related care. This effort included identifying data that are helpful during a health care visit to address the first steps in a tiered, stepwise approach to managing obesity and identifying data (eg, diet information, blood pressure, and medications) that are useful for preventing chronic diseases.

### Development and alignment

The development of Healthy Weight standards advanced through 2 independent organizations. The first organization, Health Level Seven International (HL7), is devoted to the development of interoperability standards for EHI exchange, integration, sharing, and retrieval. We defined data message content in partnership with domain experts from the American Academy of Pediatrics; technical experts from Lantana Consulting Group; members of CDC’s Healthy Weight Expert Panel, which consisted of clinical and health care management experts; state and local partners who participated in CDC’s Healthy Weight webinar; and additional content and surveillance experts. We accepted, documented, and integrated input from October 2010 through September 2013. Members of the HL7 Public Health and Emergency Response Committee wrote the implementation guide for the Healthy Weight standards. An approval process took place before publication that included a public comment period, a comment resolution involving the HL7 Public Health and Emergency Response Committee and the party who suggested the changes, and incorporation of agreed-upon revisions.

The second organization, Integrating the Healthcare Enterprise International (IHE), is a public–private global initiative supported by end users worldwide to improve patient care by harmonizing the exchange of health care information. Standards were developed through a similar process to that of HL7, including input from CDC’s expert panel, webinar participants, additional experts, and public comment. The IHE Quality Research and Public Health Committee wrote a case profile (a common language for purchasers, vendors, and developers defining how standards can be implemented to meet certain clinical needs) that specified both message content and interoperability requirements; the case profile defined actors (those responsible for producing, managing, and/or acting on information) in 5 data-flow transactions used to capture Healthy Weight data and communicate these data to public health information systems. The development of complementary standards by 2 organizations independently was intended to increase interoperability and maximize use of Healthy Weight data by information systems.

We submitted data code requests to Regenstrief Institute (www.regenstrief.org) to create new LOINC (Logical Observation Identifiers Names and Codes) data elements that could be used in health management. Additionally, we created Healthy Weight value sets (ie, groups of codes used to define clinical concepts) in conjunction with the HL7 and IHE development processes. The Healthy Weight value sets are housed in the Public Health Information Network Vocabulary Access and Distribution System (21), a system that facilitates accessing, searching, and distributing standards-based vocabularies to support the exchange of information.

Alignment activities aimed to match the content of Healthy Weight standards to existing domain guidelines and measures. Thus, Healthy Weight standards were aligned with obesity-related meaningful-use standards and incentive programs for clinical quality measures (22,23), obesity management guidelines (15,24), and use-case guidelines for pediatric Healthy Weight EHRs (6). Additionally, the Healthy Weight data elements were aligned with the core physical activity questions called for as vital signs in global initiatives (25), which can be used to assess whether or not patients meet physical activity guidelines (26,27). Similarly, these standards align with standard codes used in nutritionist referrals (9) and with EHI standards in health surveillance (28). To ensure comparability with existing information systems, the Healthy Weight standards use questions (eg, http://tools.nccor.org/measures; www.euro.who.int/en/data-and-evidence) and protocols (eg, pregnancy status and other elements needed to meet BMI data cleaning protocols) (29) from existing national and international surveys. Alignment was addressed in the early stages of developing the Healthy Weight standards to help ensure that the multiple initiatives produced complementary work products.

Alignment activities took place through a collaborative development process. Alignment promotes interoperability, which optimizes integration of similar data and maximizes use of health care information by health care providers, practitioners, advocates, and payers. Webinars hosted by CDC for state and local partners throughout the development process allowed input from leaders in EHI-based surveillance and others who planned to use the data. Federal subject matter experts provided input on topic areas such as obesity, nutrition, physical activity, and surveillance. Also, professional organizations co-led or contributed to the development of the Healthy Weight standards to ensure that the standards would meet the needs of their members. Health information system vendors engaged in this project provided expertise on integration solutions and existing technical frameworks (30,31). Additionally, CDC convened the Healthy Weight Expert Panel, who identified gaps in EHR data capture and prioritized potential Healthy Weight data elements for inclusion according to clinical and surveillance needs.

### Testing and demonstration

After creating the Healthy Weight standards, we began a testing and demonstration process to stimulate development, awareness, and dissemination. Implementers built EHI systems to adhere to the specifications defined in the Healthy Weight profile and then introduced these EHI products at several international conferences (www.ihe.net/Connectathon/; www.himssconference.org/; http://phiconference.org/). Implementers tested whether Healthy Weight messages could be exchanged between entities. After EHI systems passed testing, implementers demonstrated use-case scenarios at interoperability showcases (forums where implementers exhibit their capabilities with the purpose of spurring adoption).

## Results of the Standards Development Process

### Defined data content

Two Healthy Weight message standards, a screening version 2.5.1 observational result and an advanced clinical document architecture, were published (32,33). These standards use data elements from the “ABCDs” of a healthy weight visit that addresses the first steps of obesity prevention and care: **a**ssessment, **b**ehaviors, **c**ontinuity, i**d**entify resources, and **s**et goals (Table 1). The Healthy Weight messages use Systematized Nomenclature of Medicine (SNOMED) and LOINC to express the data elements. We created 31 LOINC codes, which filled gaps where Healthy Weight data elements did not previously exist. Each data element is defined with a level of optionality; for example, weight is a required data element, whereas waist circumference is included only if known. Thus, jurisdictions have flexibility in adapting Healthy Weight messages to local needs and requirements.

**Table 1:** The “ABCDs” of Healthy Weight and Overview of Corresponding Section and Data Elements From the Screening Standards and Advanced Healthy Weight Standards^a^

Healthy Weight Category Concept	Section	Data Elements
**A**ssessment: anthropometrics and demographics	Administrative	Demographics (eg, date of birth, sex); visit information (eg, date of service, facility, provider); payer
	Active problems	Associated conditions value set
	Vital statistics	Height and weight; expanded set of vital statistics (eg, waist circumference)
**B**ehaviors related to weight	Social history	Quantity of fruits and vegetable intake, breastfeeding, physical activity, screen time, sleep Value sets: nutrition, physical activity, breastfeeding Readiness for change Occupation, school, education
**C**ontinuity of care	Family history	Value set
	Laboratories	Value set
	Medications	Medication list
	Procedures and interventions	Interventions value set
i**D**entify resources in the community	Resources	Linking to resources (eg, additional supports in health care, personal care, public health, the community)
**S**et goals: supply a Healthy Weight care plan	Care plan	Patient-centered plan, including behavior goal setting

a Technical guides for comprehensive list of data elements are available (32,33). Screening Healthy Weight content of the version 2.5.1 observational results message standard are the data elements are noted in bold. Advanced Healthy Weight content of the clinical document architecture message standard has options for all data elements listed in the table.

The core screening content of the Healthy Weight message contains anthropometric data such as height and weight as well as data on age and sex to enable the calculation of BMI and BMI percentile. Also, this core content includes data on basic demographic characteristics, which are useful for patient care and subpopulation prevalence estimates. Additionally, it has a value set for associated conditions (21) that identifies conditions associated with or affecting changes in BMI; these data can be used in interpreting BMI data for both clinical and surveillance purposes. For example, the BMI values for a woman who is pregnant or for a person whose leg was amputated are interpreted differently by health providers than values for people without those conditions; these latter BMI values are not included in analyses of national obesity estimates (29). These screening Healthy Weight message elements are included in the message standard for the observational result (32,33).

The comprehensive advanced content in the message standard for the clinical document architecture comprises the aforementioned screening elements plus data elements for the “ABCDs” (33). These additional elements support initial steps in obesity care. Data elements prioritized from the social history section of the clinical note include an assessment of health behaviors (eg, nutrition, physical activity), patient’s readiness to change each behavior, and settings that influence behaviors (eg, school, worksite). Continuity of care elements from the sections on family history, laboratory results, medication, procedures, and interventions provide additional information to individualize and support patient care. Identification of community resources jointly by health care providers and families gives opportunities to patients for obesity prevention and treatment after the clinic visit. This Healthy Weight content is used to develop a plan for obesity-related care tailored to the behavior goals set together by the patient and the physician. The content helps to empower patients, coordinate care, conduct evaluations, and improve outcomes.

Seven Healthy Weight value sets published in the Public Health Information Network Vocabulary Access and Distribution System (21) support Healthy Weight message standards. In addition to the Healthy Weight associated conditions value set described above, these value sets include value sets for physical activity behavior, nutrition behavior, family history, laboratory tests, maternal breastfeeding, and interventions. Each value set is assigned a unique object identifier, which enables precise data storage. These object identifiers are part of the Healthy Weight messages and are referenced in the 2 technical publications (32,33).

### Scenarios for interoperability and use

**Figure 2:**
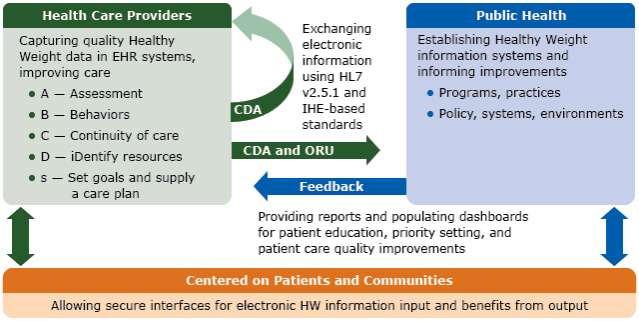
Electronic Healthy Weight information exchange process flow chart, 2015. Information flow between participants (boxes) via interactions (arrows) is enabled by standards using the Healthy Weight ORU assessment and CDA advanced message content. The “ABCDs” are captured in EHR systems in health care providers’ offices. Selected data can be securely transmitted between health care providers and public health agencies for coordination and improvement of individual and population-level care. Processed, enhanced data are shared for use in education, priority setting, and quality improvement. Abbreviations: CDA, clinical document architecture; EHR, electronic health record; HL7, Health Level Seven International; HW, Healthy Weight; IHE, Integrating the Healthcare Enterprise International; ORU, observational result; v, version.

The message standards for Healthy Weight electronic information systems facilitate the collection of data, coordination of care, and exchange of information (Figure 2). Starting in the health care providers’ offices, EHR systems can capture high-quality Healthy Weight data that support patient care. Five use-case scenarios outline the actors and transactions for interoperability between systems (33). Healthy Weight electronic information systems can receive and process data. These data have the potential to inform patient-level and systems-level improvements.

Through public–private engagement, 15 health IT implementers began developing systems that meet Healthy Weight standards, testing data flow, and demonstrating data integration scenarios. Two publications provide technical guidance for IT developers: 1)* HL7 version 2.5.1 Implementation Guide: Height and Weight Report* (32), which defines ORU message standard content, and 2) the IHE Quality Research and Public Health Technical Committee’s *Healthy Weight Technical Framework Supplement For Trial Implementation* (33), which defines ORU and CDA message content and interoperability.

An IT implementer demonstrated at interoperability showcases how EHRs can capture and track high-quality Healthy Weight information at the pediatric well-child visit to inform care, accurately document the child’s growth trajectory, and improve practice quality. This information, along with other information on routine care (eg, administration of immunizations), were securely communicated as a message to health registries, which then could send parsed information to permitted health care providers and public health groups that support patient-level and community-level health activities. Other implementers demonstrated a scenario in which an adult was seen for a weight-management visit. The “ABCDs” of Healthy Weight were available in the EHR for health care providers, thus delivering information for decisions, facilitating efficient workflow, and improving documentation. EHRs exchanged data with personal devices and health records, a waiting-room kiosk, and specialist referrals, thus providing objective data and improving care coordination. Once EHI data from multiple patients were combined and assessed, this scenario illustrated how analyzed information could be used to help prioritize a worksite intervention to strengthen the community’s obesity management efforts.

## A Roadmap for Healthy Weight Informatics

IT data standards for the Healthy Weight measure are published and available for use by all stakeholders. Common metrics and coordination across strategies could be leveraged for an intervention framework that broadens capacity, combines commitments, streamlines efforts, and unites on common measures of high-impact, high-burden health issues. Enhancements of Healthy Weight standards continue via the refinement and maintenance process. Continued harmonization through comparative analyses to relevant Healthy Weight and EHR-based efforts will identify gaps, overlaps, and recommendations for changes. In particular, advancements in the Structured Data Capture Initiative (http://wiki.siframework.org/Structured+Data+Capture+Initiative) present an opportunity for Healthy Weight standards to align with developing architectures for precise collection of data elements in EHRs. Also, Healthy Weight standards should continue to be updated with feedback from implementers and best practices as the science advances.

A roadmap guides stakeholders with potential actions steps (Table 2) for initiating, examining, and prioritizing future efforts. Movement toward fully integrated systems of care will require collective input and effort across priority action steps (7). Although establishing standards facilitates seamless information flow, strategic activities also need to address the priority areas of information systems and the contextual factors in which they function. Highlighting early successes and identifying actions may accelerate progress along this roadmap for Healthy Weight electronic information systems. An additional consideration is how work in other risk-factor assessments and chronic disease monitoring can leverage mutual advancements in this field and related fields.

**Table 2:** Categories of Strategic Activity Inputs and Expected Outcomes for Healthy Weight Standards

Category	Examples
**Strategic Activity Inputs**	
**Opportunity action level**	**Potential priority action steps for stakeholders^a^**
Data	Use common standards, measures; implement, refine, and support international health information technology standards as well as develop supporting data elements for common metrics across all stakeholder health information systems
Information systems	Develop systems infrastructure supportive of packaging, sending, and receiving data Determine the best approach to manage information
Context	Identify, develop, recommend, and implement informed policies and payments to support integrated data systems Link components into an attractive package consisting of tools from across stakeholder groups supportive of development, dissemination, and financing
**Expected Outcomes**	
**Process results**	**Example of a patient outcome and a population outcome**
Improved data capture	Better patient care delivery Better data for population analytics
Systems integration	Better patient care coordination Better linkages for health collaborations
Improved data use	Feedback supporting patient and practice quality improvements Informed community priorities, policies, and programs

a Stakeholder groups include health care providers and agencies, health information technology vendors, state and local public health professionals, patients, communities (eg, local organizations, leaders, coalitions) that are consumers of health care services and users of person-centered electronic health portals and devices, payers (ie, health insurers), lawmakers, federal agencies, and international organizations.

Healthy weight informatics outcomes can build in the short term (the next few years), intermediate term, and long term (within 10 years) to increasingly benefit individuals and communities. The ultimate goal of cross-stakeholder engagement in coordinated, strategic action is to advance the health and well-being of the population through improved prevention and management. Activities are intended to improve outcomes related to each measure of the Healthy Weight standard content. Thus, coordinated actions lead to improved BMI, behaviors, and health care satisfaction; empowered patients active in self-management; and resilient communities designed for wellness.

Healthy Weight activities fit within and support broader international strategic directions for obesity, IT, surveillance, and health care and public health linkages. The intervention framework of obesity prevention and management spans key behaviors (eg, nutrition, physical activity) and settings (eg, health care, community, day care, school, worksite) (34). Healthy Weight standards capture data on measures across these behaviors and settings to inform interventions and care. Also, CDC’s surveillance strategy (35) refers to the application of health IT and standards to improve public health surveillance. Healthy Weight standards contribute to public health surveillance by harmonizing data standards for the submission of relevant data to health departments. This information can enable decision makers to take appropriate action on obesity and monitor results. Additionally, Healthy Weight EHI efforts span integrated health care strategies (2,4) with work to enhance care by improving systems in the health care facility, providing support for the clinic visit and empowering patients, and making connections between patients and efforts in the community. The infrastructure for integrated health could be realized with Healthy Weight information systems that serve to benefit patients, health care providers, and communities.

## Conclusions

Healthy weight is a priority health concern among patients, health care providers, and communities, and addressing its magnitude calls for coordinated prevention and management efforts. Now that the Healthy Weight standards have been developed, aligned, and tested, they are ready for use. Collaborative multi-stakeholder activities that use health IT and leverage standards across systems have the potential to improve information systems and accelerate positive outcomes. In the patient–provider interaction, capturing and fully using standardized Healthy Weight data may transform the delivery and coordination of care, increase the quality of and satisfaction with obesity-related care, decrease costs, and improve health outcomes. At the public health or system level, accessing and maximizing Healthy Weight data may fill gaps in surveillance and population-level analytics, inform evaluations and interventions, and ensure improvements to build healthy communities. Prioritizing action steps and synergistic efforts across stakeholders may catalyze achievement of positive outcomes and thus advance global health and well-being.
